# Clinical outcomes and risk factors of *Acinetobacter baumannii* meningitis in pediatric patients at a tertiary hospital in China

**DOI:** 10.3389/fcimb.2024.1408959

**Published:** 2024-08-29

**Authors:** Zixuan Wang, Lijing Ye, Pan Fu, Xia Wu, Jun Xu, Yingzi Ye, Shuzhen Han, Chuanqing Wang, Hui Yu

**Affiliations:** ^1^ Department of Infectious Diseases, Children’s Hospital of Fudan University, National Children’s Medical Center, Shanghai, China; ^2^ Lab of Microbiology, Department of Clinical Laboratory, Children’s Hospital of Fudan University, National Children’s Medical Center, Shanghai, China

**Keywords:** *Acinetobacter baumannii*, meningitis, carbapenem-resistant, clinical characteristics, outcomes, children

## Abstract

**Objectives:**

To summarize the clinical characteristics, outcomes and identify risk factors of *Acinetobacter baumannii* (AB) meningitis in children.

**Methods:**

This was a single-center, retrospective study. Children hospitalized between January 2016 and December 2021 who were diagnosed with AB meningitis were included. The clinical characteristics and outcomes were reviewed. Risk factors were determined using univariate analyses (chi-square and Mann-Whitney U tests).

**Results:**

Seventeen patients were included; 15 cases were secondary to neurosurgery, and two were neonates with primary bacterial meningitis. Common symptoms included fever, convulsions and nervous system abnormalities. Cerebrospinal fluid (CSF) tests typically showed increased white blood cell counts dominated by neutrophils, reduced glucose levels and elevated protein levels. Ten patients were successfully treated (successful treatment [ST] group); seven had failed treatment (failed treatment [FT] group). Univariate analyses revealed that mechanical ventilation, routine white cell counts in the peripheral blood, procalcitonin, protein in the CSF, septic shock and carbapenem-resistant AB (CRAB) differed significantly between the groups.

**Conclusion:**

AB meningitis in children has a high mortality rate. FT was associated with mechanical ventilation, septic shock, CRAB, lower peripheral leukocyte counts, higher protein levels in the CSF and procalcitonin. Larger studies are needed to identify independent risk factors for adverse outcomes.

## Introduction

*Acinetobacter baumannii* (AB), a gram-negative coccobacillus, is an opportunistic pathogen. It is primarily associated with patients admitted to hospitals or undergoing healthcare-related treatments, particularly those who receive treatment in intensive care units (ICUs). AB can cause multiple system infections, including pneumonia, bloodstream infections, meningitis, skin infections and soft tissue infections.

Bacterial meningitis is among the most difficult diseases to treat in children and exhibits a poor prognosis. AB is a significant nosocomial meningitis agent (Chusri et al., 2018) and accounts for >25% of all pathogens isolated from cerebrospinal fluid (CSF) (Chen et al., 2021).However, the resistance rates of AB to various antibiotics continue to increase yearly. Few antibacterial drugs are available to treat such bacterial infections, presenting great therapeutic challenges (Tamma et al., 2022). The current treatments are mainly combing at least two antibiotics based on tigecycline, polymyxin B, sulbactam or carbapenems (Tamma et al., 2023). Bacterial meningitis with extensively drug-resistant pathogens can lead to ineffective antibacterial treatment and has the highest mortality rates among infections (Mauldin et al., 2010). A previous study reported that AB-induced central nervous system (CNS) infections account for a high risk of death in pediatric patients (Shi et al., 2020). AB meningitis is rare in children. The lack of clinical cases, nonspecific clinical manifestations, limited drugs choices, and poor immunity in children increase the severity of the damage caused by this infection and the challenges of clinical treatment (Ye et al., 2020).

AB meningitis often occurs secondary to neurosurgery, leading to high mortality rates and neurological sequela (Chusri et al., 2018). Most previous studies have focused on the risk factors for the occurrence of AB meningitis, such as neurosurgery, head trauma, CSF leakage, wound infection, and foreign body implantation (Korinek et al., 2006). However, few studies have focused on the risk factors for poor outcomes.

Carbapenem-resistant AB (CRAB) has become increasingly problematic in recent years (Kyriakidis et al., 2021). A previous systematic review reported that CRAB is a leading pathogen associated with deaths attributable to bacterial antimicrobial resistance and caused more than 50000 deaths in 2019. Furthermore, CRAB was the fourth leading pathogen-drug combination globally for 2019 (Antimicrobial Resistance, 2022). Patients with CRAB have double the mortality rate and a significantly greater burden of illness compared with those with carbapenem-susceptible AB (CSAB) (Lemos et al., 2014; Pogue et al., 2022). Multiple studies have shown that CRAB was the most common pathogen in non-survivors of bacterial meningitis, and the worst clinical outcomes often occurred with CRAB infections (Shi et al., 2020; Thatrimontrichai et al., 2021; Panic et al., 2022; Zheng et al., 2022). However, few studies have compared the differences in clinical manifestations and outcomes between meningitis patients with CRAB vs CSAB.

Few reports have been published on AB meningitis in children, and of these, few were case reports or reviews. Therefore, we retrospectively analyzed children with this infection who were hospitalized at our institution. Our primary objective was to summarize the clinical characteristics and outcomes and identify key factors associated with the infection, thereby enhancing clinicians’ understanding and management of this serious condition.

## Methods

### Setting and study design

This retrospective study was conducted in the Children’s Hospital of Fudan University, a national children’s medical center. Children (aged ≤18 years) hospitalized from 1^st^ January 2016 to 31^st^ December 2021 from whom AB was detected in the CSF were included. Only the first episodes of positive cultures was included to avoid case duplication.

The inclusion criteria were (1) isolation of AB from the CSF; (2) increased white blood cell counts (≥10×10^6^/L) and protein levels (≥450 mg/L) and decreased glucose in the CSF; and (3) clinical evidence of bacterial meningitis (i.e., fever, headache, vomiting, confusion, irritability and meningeal irritation). The exclusion criteria were (1) incomplete medical records and (2) the cases in which positive CSF cultures were obtained in the absence of clinical and laboratory features of meningitis.

Patients were divided into either the successful treatment (ST) or failed treatment (FT) group according to patient outcomes. ST was defined as two consecutive negative CSF cultures with clinical symptoms that had disappeared and normal routine peripheral blood and CSF tests. FT was defined as persistent positive CSF cultures before discharge or patient’s eventual death.

Patients were also divided into CRAB group and CSAB group according to results of susceptibility testing. And we compared clinical characteristics between the two groups.

### Data collection

Medical records of all included patients were reviewed. Data extracted included demographic information, hospital stay length, hospitalization ward, days from first neurosurgery to diagnosis, clinical features, diagnosis, underlying diseases, previous hospital admission within 6 months, invasive procedures, and receipt of immunosuppressive therapy (including cytotoxic agents within 6 weeks or corticosteroids at a dosage ≥10 mg of prednisolone daily for >5 days within 4 weeks prior to detection (Chusri et al., 2019). Additional data included antibiotic resistance, previous antibiotic therapy within 30 days, co-infections with other bacteria or fungi, antibiotic treatment, time to CSF sterilization, and patient outcomes. Sepsis and septic shock were evaluated by clinician according to the Sepsis-3 (Singer et al., 2016). And neonatal sepsis was evaluated by clinician according to the consensus of domestic experts (Subspecialty Group of Neonatology, t. S. o. P. C. M. A and Professional Committee of Infectious Diseases, N. S. C. M. D. A, 2019).

### Antibiotic resistance and antimicrobial susceptibility testing

We reviewed the antibiotic-resistance profiles of included AB isolates. Matrix-assisted laser desorption ionization-time of flight mass spectrometry (MALDI-TOF/MS) (Bruker Daltonics, Bremen, Germany) was used to confirm the species of the isolates. AST was performed in the microbiology laboratory of the hospital using the Kirby-Bauer method or automated systems (VITEK 2 Compact). AST was conducted for 14 agents: imipenem, meropenem, levofloxacin, amikacin, gentamicin, ceftazidime, cefepime, piperacillin-tazobactam, ampicillin-sulbactam, cefoperazone-sulbactam, colistin, minocycline, tigecycline and sulfamethoxazole-trimethoprim (TMP-SMX). Antibiotic susceptibilities were defined according to the criteria of the Clinical and Laboratory Standards Institute, 2022 breakpoints (Clinical and Laboratory Standards Institute, 2022). CRAB was defined as having minimum inhibitory concentrations (MICs) of imipenem or meropenem ≥8 mg/L. CSAB was defined as having MICs of imipenem and meropenem ≤2 mg/L.

### Statistical analyses

Categorical data were summarized as counts and percentages; chi-square and Fisher’s exact tests were used for comparisons between two groups. Continuous data were presented as means ± standard deviation or median with interquartile range (IQR), depending on the degree of skewness in the distributions evaluated using the Shapiro-Wilk test. Differences were identified using t-tests or Mann-Whitney U tests. *P*<0.05 was considered statistically significant. Statistical analyses were performed using SPSS statistical software, version 23.0.

## Results

### Patients’ clinical characteristics

Seventeen patients (9 boys; 8 girls) with AB meningitis hospitalized from 1^st^ January 2016 to 31^st^ December 2021 were included. **Table 1** showed their clinical characteristics. The median age of the patients was 24.00 months (IQR, 17.00–107.00 months); only one patient was a neonate. Ten patients (58.82%) were in the ICU; seven (41.18%) were in general wards. Of the 17 patients, 15 were cases secondary to neurosurgery; two were neonates with primary bacterial meningitis. The average time from the first surgery to positive CSF culture was 15.00 days (IQR, 4.50–24.00 days). All patients had underlying diseases, including craniocerebral tumor (12% [70.59%]), intracranial hemorrhage (1 [5.88%]), hydrocephalus (2 [11.76%]) and neonatal sepsis (2 [11.76%]).

**Table 1:** Characteristics of patients with *Acinetobacter baumannii* meningitis.

Characteristics	All patients (N=17)	CRAB (N=12)	CSAB (N=5)	*P-*value
Male	9(52.94%)	6(50.00%)	3(60.00%)	1.000
Age, m, median (IQR)	24.00(17.00,107.00)	23.50(17.00,142.75)	28.00(9.49,50.00)	0.598
Weight, kg, median (IQR)	16.88 ± 12.51	19.01 ± 14.01	11.78 ± 6.38	0.165
Length of hospital stay, d, median (IQR)	91.65 ± 62.10	102.42 ± 67.82	65.80 ± 39.96	0.191
Hospitalization ward				
Pediatric intensive care unit	10(58.82%)	10(83.33%)	0(0.00%)	0.003
General wards	7(41.18%)	2(16.67%)	5(100.00%)	
Clinical features of infections				
Duration of fever, d, median (IQR)	20.12 ± 10.51	22.25 ± 11.37	15.00 ± 6.28	0.204
White cell count, ×10^9/L, median (IQR)	17.00(12.95.24.75)	16.00(12.55,28.70)	17.00(12.65,20.90)	0.527
Neutrophil, %, median (IQR)	76.70(61.05,83.00)	81.05(62.70,83.55)	68.90(60.85,75.15)	0.154
C-reactive protein, mg/L, median (IQR)	87.80 ± 51.23	78.22 ± 53.40	110.80 ± 41.46	0.244
Procalcitonin, ng/ml, median (IQR)	1.03(0.11,2.92)	1.13(0.19,3.30)	0.14(0.06,2.18)	0.141
Interleukin 6, pg/ml, median (IQR)	126.60(41.78,262.60)	235.70(126.60,283.90)	45.02(38.55,46.30)	0.087
Sepsis	6(35.29%)	5(41.67%)	1(20.00%)	0.600
Septic shock	7(41.18%)	7(58.33%)	0(0.00%)	0.044
White cell count in CSF,×10^6/L, median (IQR)	1160.00(495.00,2485.00)	1200.00(625.00,2492.50)	1000.00(170.00,1830.00)	0.205
Protein in CSF, mg/L, median (IQR)	3577.90(2134.10,5755.00)	4744.20(3236.73,7500.90)	2306.50(1134.30,2643.45)	0.011
Glucose in CSF, mmol/L, median (IQR)	0.80(0.05,1.85)	0.10(0.00,1.50)	1.60(1.15,2.35)	0.071
Underlying condition				0.010
Craniocerebral tumor	12(70.59%)	11(91.67%)	1(20.00%)	
Intracranial hemorrhage	1(5.88%)	0(0.00%)	1(20.00%)	
Hydrocephalus	2(11.76%)	0(0.00%)	2(40.00%)	
Neonatal sepsis	2(11.76%)	1(8.33%)	1(20.00%)	
Neurosurgery	15(88.24%)	11(91.67%)	4(80.00%)	0.515
Days from first neurosurgery to diagnosis, d, median (IQR)	15.00(4.50,24.00)	10.50(4.25,24.00)	20.00(7.50,31.50)	0.527
Duration of targeted antibiotic treatment, d, median (IQR)	30.00(18.50,42.00)	34.00(14.75,42.00)	23.00(20.00,34.00)	0.597
CSF sterilization				
Yes	10(58.82%)	5(41.67%)	5(100.00%)	0.044
No	7(41.18%)	7(58.33%)	0(0.00%)	

CSF, cerebrospinal fluid; CRAB, carbapenem-resistant *Acinetobacter baumannii*; CSAB, carbapenem-susceptible *Acinetobacter baumannii*.

All patients had clinical manifestations of recurrent fever and disturbance of consciousness. 11 patients had vomiting; five had convulsions, and three were positive for meningeal irritation. During treatment, five patients developed complications: four had hydrocephalus, and one had subdural effusion. Routine peripheral blood tests showed increased white blood cell counts dominated by neutrophils, c-reactive protein (CRP) levels averaging 87.80 ± 51.23 mg/L and increased procalcitonin (PCT) with an average of 1.03 (IQR, 0.11–2.92) ng/mL. The CSF changed typically: increased white blood cell counts with an average of 1160.00×10^6^/L (IQR, 495.00–2485.00) dominated by neutrophils; decreased glucose with an average of 0.80 mmol/L (IQR, 0.05–1.85), and increased protein contents averaging 3577.90 mg/L (IQR, 2134.10–5755.00). Six patients (35.29%) had sepsis; seven (41.18%) developed septic shock.

Twelve patients had CRAB; five had CSAB according to antibiotic resistance. We compared clinical characteristics between them. Hospitalization ward (*P*=0.003) and distribution of underlying conditions (*P*=0.010) differed significantly between them. Among CRAB patients, 11 (91.67%) had craniocerebral tumors, whereas only one CSAB patient (20.00%) had a craniocerebral tumor. Protein in the CSF and the proportion of septic shock were significantly higher in the CRAB group than in the CSAB group. Conversely, the proportion of CSF sterilization was significantly lower in the CRAB group than in the CSAB group.

### AST

**Figure 1:**
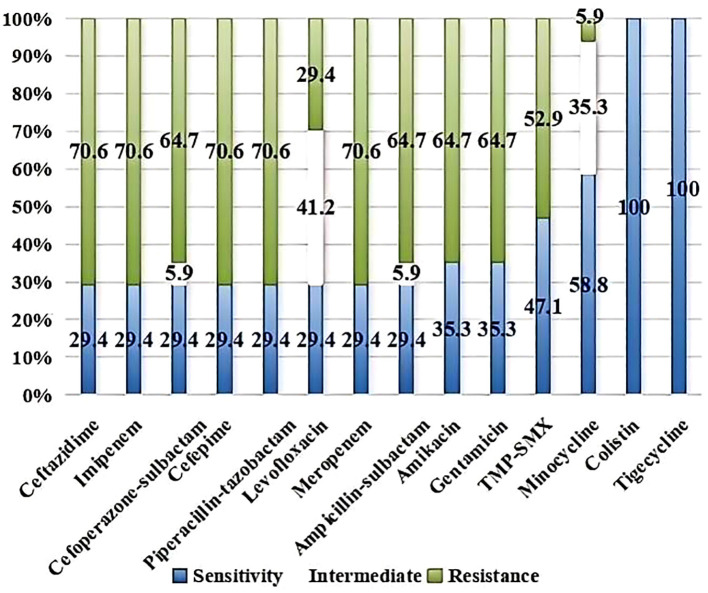
AST of *Acinetobacter baumannii*.

**Figure 1** showed the AST results of the AB isolates to common clinical antibiotics. Of these, 70.6% of the strains were resistant to meropenem and imipenem. The resistance rates to ceftazidime, cefepime, piperacillin-tazobactam, ampicillin-sulbactam, cefoperazone-sulbactam, amikacin, gentamicin and TMP-SMX were 70.6%, 70.6%, 70.6%, 64.7%, 64.7%, 64.7%, 64.7% and 52.9%, respectively. The resistance rates were lower to levofloxacin (29.4%) and minocycline (5.9%) than to other antibiotics, whereas the intermediate rates were higher (41.2% and 35.3%, respectively). No isolates that were resistant to colistin or tigecycline.

### Treatment and outcomes

Two patients had neonatal sepsis. Four patients received antibiotics to prevent perioperative infection and 11 cases underwent anti-infective treatment before being diagnosed with meningitis. When the CSF culture was positive, the treatment plan was actively adjusted according to antibiotic susceptibility. Patients with CSAB were treated primarily with dual antibiotic therapy based on meropenem, and other antibiotics included ampicillin-sulbactam, cefoperazone-sulbactam, amikacin, and fosfomycin. Of the patients with CRAB, three received dual antibiotic therapy, including meropenem and ampicillin-sulbactam, levofloxacin and sulbactam sodium, ampicillin-sulbactam and polymyxin B. Seven received triple antibiotic therapy based on meropenem and tigecycline; other antibiotics included fosfomycin, quinolones and sulbactam. The remaining two patients eventually received quadruple antibiotic therapy (meropenem, sulbactam sodium, polymyxin B and levofloxacin/fosfomycin). Three patients received intrathecal injections of polymyxin B. The average time for targeted antibiotic treatment was 30.00 days (IQR, 18.50–42.00), lasting 2–4 weeks after two consecutive negative CSF cultures. Finally, ten patients’ CSF cultures became negative. Seven patients died; all of whom had CRAB, and their CSF cultures remained positive.

### Univariate analysis of patients with AB meningitis, stratified by outcome

Of the seventeen patients included in the final analysis, 10 had ST, and seven had FT. Univariate analyses revealed that mechanical ventilation (*P=*0.044), routine white blood cell counts in the peripheral blood (*P*=0.031), PCT (*P*=0.045), protein in the CSF (*P*=0.032), septic shock (*P*=0.004) and CRAB (*P*=0.044) differed significantly between the groups (**Table 2**).

**Table 2:** Univariate analysis of factors associated with the FT group compared with those of the ST group.

Characteristic	FT N=7	ST N=10	*P* value
Age, m, median (IQR)	70.00(20.00,146,00)	22.00(12.26,39.00)	0.143
Male sex	3(42.86%)	6(60%)	0.637
Duration of hospital stay before detected, d, median (IQR)	27.00(12.00,53.00)	19.00(6.75,30.00)	0.329
Previous hospital admission within 6 m	2(28.57%)	7(70.00%)	0.153
Neurosurgery	7(100.00%)	8(80.00%)	0.485
Days from first neurosurgery to diagnosis, d, median (IQR)	9.00(5.00,25.00)	17.50(2.25,24.00)	0.845
Mechanical ventilation	7(100.00%)	5(50.00%)	0.044
Indwelling central venous catheter	6(85.71%)	5(50.00%)	0.304
Indwelling ventricular drainage	4(57.15%)	3(30.00%)	0.644
Receiving immunosuppressive therapy	1(14.29%)	0(0.00%)	0.412
Co-infections with other bacteria or fungi	2(28.57%)	4(40.00%)	1.000
Intrathecal injection	0(0.00%)	3(30.00%)	0.228
Previous antibiotic therapy within 30 d			
Cephalosporins	7(100.00%)	8(80.00%)	0.485
β-Lactam-β-Lactamase Inhibitor	4(57.15%)	2(20.00%)	0.162
Carbapenems	6(85.71%)	5(50.00%)	0.304
Vancomycin	4(57.15%)	7(70.00%)	0.644
Clinical features of infections			
Duration of fever, d, median (IQR)	19.90 ± 10.01	20.43 ± 12.00	0.923
White cell count, ×10^9/L, median (IQR)	14.85 ± 4.45	25.34 ± 9.88	0.031
Neutrophil, %, median (IQR)	82.30(76.70,86.10)	69.70(58.60,80.70)	0.107
C-reactive protein, mg/L, median (IQR)	76.87 ± 48.31	103.43 ± 54.90	0.308
Procalcitonin, ng/ml, median (IQR)	1.87(1.07,4.36)	0.14(0.90,2.18)	0.045
Interleukin 6, pg/ml, median (IQR)	47.58(38.55,186.10)	47.58(32.07,235.70)	0.210
White cell count in CSF,×10^6/L, median (IQR)	1200.00(1000.00,2470.00)	1080.00(198.75,2500.00)	0.463
Protein in CSF, mg/L, median (IQR)	4960.00(3577.90,7980.20)	2440.25(1415.48,4541.00)	0.032
Glucose in CSF, mmol/L, median (IQR)	0.10(0.00,1.70)	1.20(0.18,2.15)	0.184
Sepsis	5(71.43%)	2(20.00%)	0.058
Septic shock	6(85.71%)	1(10.00%)	0.004
Hospitalization ward			0.134
Pediatric intensive care unit	6(85.71%)	4(40.00%)	
General wards	1(14.29%)	6(60.00%)	
Detection with CRAB	7(100.00%)	5(50.00%)	0.044
Duration of targeted antibiotic treatment, d, median (IQR)	17.00(13.00,42.00)	34.00(22.25,42.00)	0.186

ST, successful treatment group; FT, failed treatment group; CRAB, carbapenem-resistant *Acinetobacter baumannii*; CSF, cerebrospinal fluid.

## Discussion

AB has become a major problematic pathogen in healthcare settings for extensive drug resistance. All-cause mortality from *Acinetobacter* spp. meningitis is reported to range from 15%–71%, and the highest mortality rates have been observed in neonates (Kim et al., 2009). Children have weaker immunity; thus, when infection occurs, the harm is more serious. Therefore, special attention should be paid to AB meningitis.

AB meningitis is mostly secondary to neurosurgery (Xiao et al., 2019). In our study, 15/17 patients (88.24%) underwent neurosurgery. Owing to a lack of specificity of clinical manifestations, clinicians often have difficulty identifying AB meningitis in time (Saleem et al., 2011). Most patients presented with fever, convulsions, and nervous system abnormalities. Blood routine examinations showed that inflammatory indicators, including CRP, PCT and interleukin-6, were significantly increased. CSF tests typically showed increased white blood cell counts dominated by neutrophils, reduced glucose levels and significantly elevated protein levels.

Because the safety of children’s medication is crucial, the choice of antibiotics is greatly limited. The guidance by Infectious Diseases Society of America (IDSA) recommended combining at least two active antibiotics when possible to treat AB infection (Tamma et al., 2023). For first-line treatment, meropenem is the most commonly recommended empiric treatment (Tunkel et al., 2017). In our study, patients with CSAB were treated mainly with meropenem. However, its resistance rate exceeds 70%. In addition, CRAB strains were resistant to most antibiotics except polymyxins and tigecycline. For these strains, guidelines recommend administering polymyxin B intravenously or in combination with intrathecal injection to treat CRAB meningitis (Tunkel et al., 2017; Chinese Research Hospital Association Of Critical Care, M et al., 2019). Previous literature showed for extensively drug-resistant AB meningitis, patients treated with intravenous and intrathecal/intracerebral ventricle injection of polymyxin B had a significantly lower 28-day mortality (55.26% vs. 8.70%, *P* = 0.01) and higher rates microbiological clearance (91.30% vs. 18.42%, *P* < 0.001) compared with patients treated with other antibiotics (Pan et al., 2018). In our study, two patients received intrathecal injections of polymyxin B and one patient received intravenous polymyxin B in combination with intrathecal injection. They were all treated successfully. Although CRAB is usually resistant to carbapenems, clinicians still try to combine carbapenems with other drugs to treat CRAB infections clinically. Studies have shown that high-dose, prolonged infusions of meropenem combined with other sensitive antibiotics have synergistic effects (Yu et al., 2018; Zhou et al., 2021). Therefore, among twelve patients with CRAB infections, all but two were treated with meropenem and eight cases received high-dose, prolonged infusions. However, this approach did not have a favorable outcome. Only five patients treated with meropenem were eventually cured. Among them, three cases were also treated with intrathecal injection of polymyxin B, and two cases were treated with ampicillin-sulbactam. Therefore, successful treatment did not seem to be attributable to meropenem alone. Tigecycline is another first-line antibiotic for treating CRAB; however, its concentration in the CSF is limited (Rodvold et al., 2006). Therefore, tigecycline has limited role in CRAB meningitis (Ye et al., 2020). Seven of our patients received therapy with meropenem and tigecycline, and other antibiotics included fosfomycin, quinolones or sulbactam. However, treatment ultimately failed in six of them. Therefore, clinicians must better understand CRAB treatment, and rational selection of antibacterial drugs is crucial for treating meningitis. Additionally, sulbactam is also an important option for the treatment of CRAB infections. The guidance by IDSA suggested high-dose ampicillin-sulbactam as a component of combination therapy for CRAB, regardless of whether susceptibility has been demonstrated (Tamma et al., 2023). In our study, seven patients received therapy with sulbactam. When CRAB infections were refractory to other antibiotics or in cases where intolerance or resistance to other agents, cefiderocol may also be an option (Pintado et al., 2023; Tamma et al., 2023).

In our study, 10 patients (58.82%) were ST, and seven (41.18%) were FT. Few studies have investigated the differences between FT and ST. Chen et al. reported that for nosocomial meningitis and patients with multidrug-resistant gram-negative bacteria, significantly different factors in ST vs FT included pathogen type, highest body temperature in the first 24 h of symptoms, CSF glucose level and meropenem susceptibility (for AB) (Chen et al., 2020). In our study, univariate analyses revealed that mechanical ventilation, routine white blood cell counts in the peripheral blood, PCT, protein in the CSF, the proportion of septic shock and CRAB differed significantly between the ST and FT groups. Standard laboratory parameters, such as peripheral leukocyte counts, glucose levels and protein in the CSF were not reliable predictors for infection in patients with healthcare-associated meningitis (Tunkel et al., 2017). However, in this study, patients with FT had lower peripheral leukocyte counts and higher CSF protein levels than did those with ST (*P*=0.031, 0.032). These laboratory parameters can be used as indicators to evaluate the disease severity, and these indicators should draw clinicians’ attention to these patients.

All patients in the FT group had CRAB. Due to the limited antimicrobial drugs available, CRAB tends to cause severer infections and has a worse prognosis than CSAB (Pogue et al., 2022). However, we asked whether the clinical manifestations differed between these two groups and compared their clinical characteristics. CRAB infections mostly occurred in the ICU, whereas CSAB infections mostly occurred in the general ward, consistent with the results of previous studies (Zhang et al., 2021). The widespread prevalence of CRAB in ICUs is usually related to the large number of severe patients with low immunity, catheter and mechanical ventilation and wide antibiotic use (Kyriakidis et al., 2021). The proportion of CSF sterilization was significantly lower in the CRAB group than in the CSAB group, which was understandable given the limited selection of effective antibiotics for CRAB. Notably, protein levels in the CSF were significantly higher in the CRAB group than in the CSAB group. We speculated whether this was related to the virulence differences between CRAB and CSAB but have found no other studies reporting similar results; thus, the clinical significance of our results requires further exploration.

This study had some limitations. First, the major limitation was the single-center, retrospective nature of the study. Collecting complete data was difficult and may have led to biased results. Moreover, no independent risk factor analysis was performed owing to the limited number of cases. Second, because the CRAB epidemiology differs regionally and may also vary over time in the same region, conclusions from our study may not be universally applicable to other centers.

In conclusion, AB meningitis in children was mostly secondary to neurosurgery and had a high mortality rate. FT was associated with mechanical ventilation, septic shock, CRAB, lower peripheral leukocyte counts, higher protein in the CSF and PCT. Larger studies are needed to identify independent risk factors for adverse outcomes. Because of the emergence of CRAB, choosing the best antibiotics remains challenging and critical to patient outcomes.

## Data Availability

The original contributions presented in the study are included in the article/supplementary material. Further inquiries can be directed to the corresponding author.
